# Diagnostic Utility of Radiomics in Thyroid and Head and Neck Cancers

**DOI:** 10.3389/fonc.2021.639326

**Published:** 2021-07-07

**Authors:** Maryam Gul, Kimberley-Jane C. Bonjoc, David Gorlin, Chi Wah Wong, Amirah Salem, Vincent La, Aleksandr Filippov, Abbas Chaudhry, Muhammad H. Imam, Ammar A. Chaudhry

**Affiliations:** ^1^ Amaze Research Foundation, Department of Biomarker Discovery, Anaheim, CA, United States; ^2^ Department of Diagnostic and Interventional Radiology, City of Hope National Medical Center, Duarte, CA, United States; ^3^ Florida Cancer Specialists, Department of Oncology, Orlando, FL, United States

**Keywords:** radiomics, head and neck cancer, thyroid cancer, imaging biomakers, immunotherapy resistance

## Abstract

Radiomics is an emerging field in radiology that utilizes advanced statistical data characterizing algorithms to evaluate medical imaging and objectively quantify characteristics of a given disease. Due to morphologic heterogeneity and genetic variation intrinsic to neoplasms, radiomics have the potential to provide a unique insight into the underlying tumor and tumor microenvironment. Radiomics has been gaining popularity due to potential applications in disease quantification, predictive modeling, treatment planning, and response assessment – paving way for the advancement of personalized medicine. However, producing a reliable radiomic model requires careful evaluation and construction to be translated into clinical practices that have varying software and/or medical equipment. We aim to review the diagnostic utility of radiomics in otorhinolaryngology, including both cancers of the head and neck as well as the thyroid.

## Introduction

Head and neck cancer (HNC) malignancies include cancers within the upper aerodigestive tract – anatomically including cancers of the mucosal linings of the sinuses and air pathways from the thoracic inlet up to the skull base (1). This group of malignancies is the seventh most common cancer worldwide and the ninth most common cancer within the United States (1). Considering the various anatomical regions pertaining to HNC, cutaneous neoplasms of the head and neck (e.g. melanoma, cutaneous squamous cell carcinomas, basal cell carcinomas, etc.) are not discussed in this article. Instead, malignant neoplasms of the thyroid often present with similar clinical symptoms as head and neck cancers, and both are often managed initially by otorhinolaryngologists. The goal of this review is to illustrate the diagnostic utility the field of radiomics can offer in differentiating pathology at the nascent setting of presentation.

Radiomics - “radi” deriving from the science of radiology and “-omics” to indicate mapping of the human genome (2–4) - is a rapidly evolving field that aims to provide non-invasive ability to comprehensively characterize tissues and organs from features extracted from standard-of-care medical imaging (5), including techniques such as computed tomography (CT), positron emission tomography (PET), magnetic resonance imaging (MRI), and so on. It is important to further explore the implications and significance of the clinical knowledge deduced from radiological imaging to potentiate developing a radiomic pipeline that allows for improving diagnosis development and clinical decision making when treating cancer.

Technological advancements in computer hardware and artificial intelligence enable an integrative analysis of clinical, radiomic, and bio-genomic data for cancer discovery (6–9). In the case of radiomics, vast numbers of quantitative features can be derived from multi-modal medical images using computational methods (3, 10). Phenotypes represented using radiomic features may have prognostic and diagnostic value, and potentially improve clinical decision support in cancer treatment (6, 11, 12).

**Figure 1:**
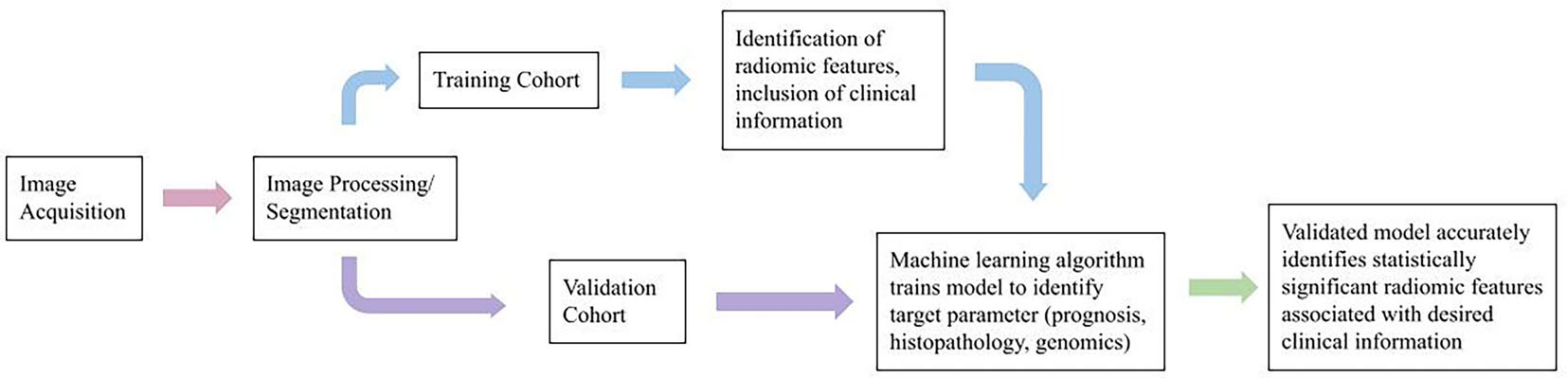
Typical radiomic workflow.

Radiomics can be performed using multimodal (CT, PET, MRI, and ultrasound) and/or multiparametric (multiple MRI sequences, e.g., diffusion MRI, perfusion MRI techniques (7–9, 13–15). In a typical radiomic workflow (**Figure 1**), we first perform image registration and pre-processing, then image segmentation and annotation. Next, radiomic features are calculated using computational methods. A variety of tools are available to streamline the process (16–24). Radiomic features are mostly sub-visual and can be coarsely grouped into intensity, shape, and texture. In addition, before calculating the radiomic values, we can apply spatial filters such as wavelets and Laplacian of Gaussian filters to extract a variety of derivative and spatial-frequency information.

The radiomic features are then integrated with other data sources for prognostic (7–9, 25–39), treatment response (40–42), histopathological (43–48), or radiogenomic (11, 49–51) analyses using statistical or machine learning modeling techniques.

## Head and Neck Cancer

**Figure 2:**
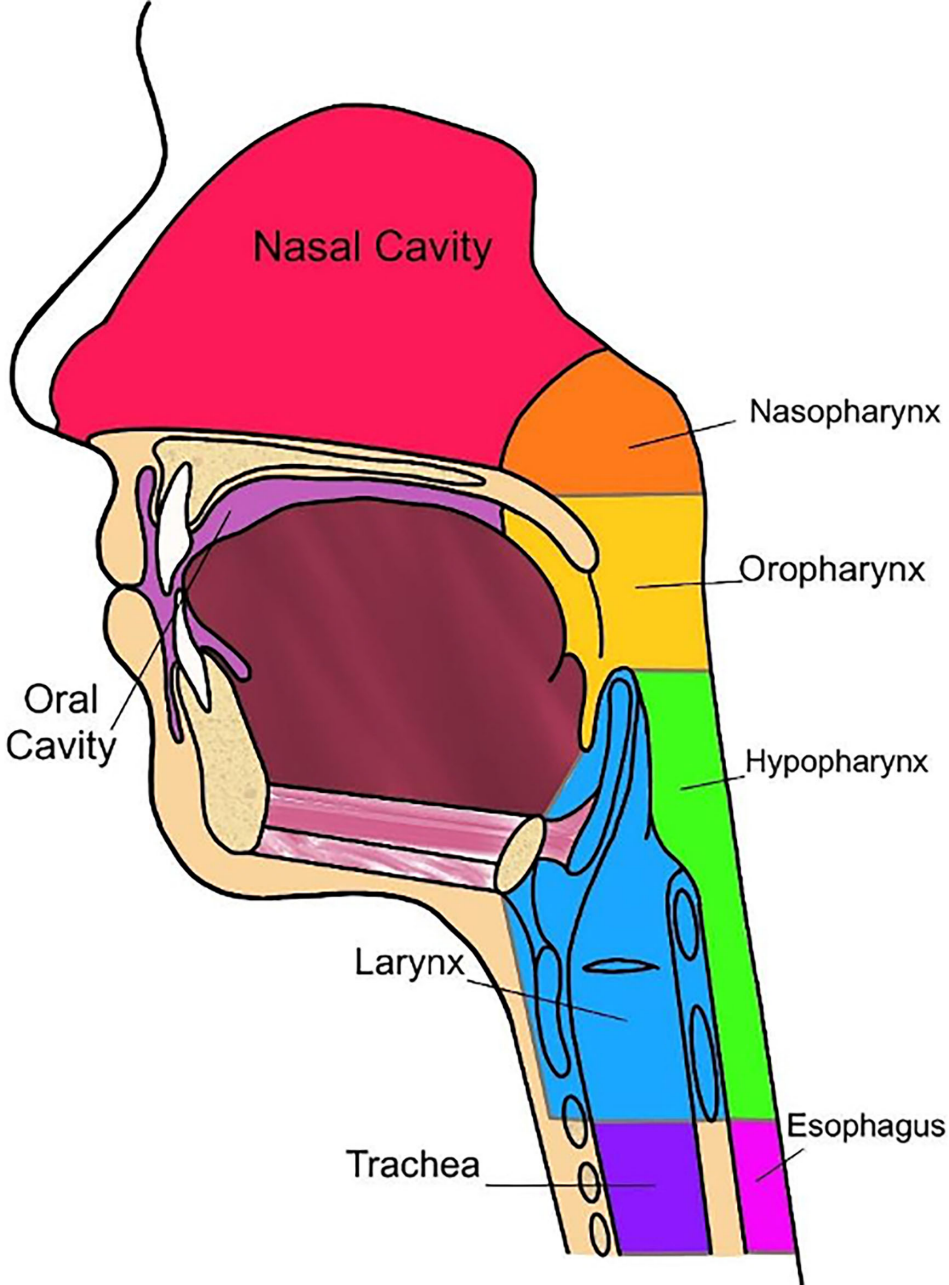
Anatomy of ear, nose, and throat, sagittal view.

Oncologic disease developing in the mucosal surfaces of anatomic subsites, such as the nasopharynx, oropharynx, hypopharynx, oral cavity, larynx, paranasal sinuses, and salivary glands are considered HNC (**Figure 2**) (52, 53). The International Classification of Diseases, Tenth Revision (ICD-10) reports that oral and pharyngeal cancer accounts for approximately 2.3% of cancers within the United States. Oral and pharyngeal cancer has a five-year survival of 27.8% and is internationally considered to be the sixth most common cancer (54, 55). Risks of developing this disease are commonly associated with the consumption of tobacco and alcoholic products. Therefore, 74% of the general population that practice tobacco and alcohol consumption have a greater risk of developing oral and pharyngeal cancer, with an estimated 80% of that population being male and 61% being female (54).

Research has also indicated an etiological association of head and neck cancer to viruses (56). The human papillomavirus (HPV), a virus known to cause common conditions such as warts, has developed a reputation for its association with cervical and oropharyngeal cancers (53). Therefore, when diagnosing HNC, patients will often be screened for HPV infection as a potential cause of disease. There are over 170 different types of HPV’s, categorized by the virus’s characteristics such as location (mucosal or cutaneous anatomical sites), response to an external stimulus, and its risk for malignancy. The mucosal subgroup of HPV is primarily associated with HNC as this subgroup contains over 40 subtypes that are considered to be sexually transmitted diseases (STD) and predominantly infect the reproductive and respiratory tracts (53).

Additional etiological associations to HNC include the Epstein-Barr virus (EBV), which is often associated with many different types of human cancers, including those of lymphoid and epithelial cells (57). Considered one of the most common human viruses, EBV infection typically spreads undetected and can reside within the host over a span of ages in which infection is dependent on several factors such as genetic predisposition, diet, living conditions, hygiene, and sexual behavior (53, 58). To further validate the commonality of EBV infection, statistics show by adulthood approximately 90-95% of the population will sustain a permanent, asymptomatic infection of EBV (53, 57). As a member of the *Herpesviridae* family, alternatively known as human herpesvirus type 4 (HHV4) (58), post-primary infection of EBV is permanent and can subsequently result in the virus shedding into genital and salivary secretions that increase the risk of carcinogenesis into HNSSC.

Currently, radiomics can predict some tumoral characteristics linked to patient survival in HNC (**Table 1**). In a study performed by Mukherjee et.al., radiomic features were analyzed *via* CT imaging to non-invasively predict the histopathological features of HNSCC. This study was performed retrospectively, utilizing CT images and data from clinically diagnosed patients with HNSCC. An institutional test cohort (n = 71) and an HNSCC training cohort derived from The Cancer Genome Atlus (TCGA) (n = 113) were analyzed within this study (43). A machine learning model, trained with 2,131 extracted radiomic features that were utilized to predict tumor histopathological characteristics, was applied to the training and test cohort. These features included intensity, size and shape, texture, and filters (43). The cancer characteristics investigated related to these features were tumor grade, perineural invasion, lymphovascular invasion, extracapsular spread, and HPV status (p16 expression) (43). For dimensionality reduction and classification of these features, principal component analysis, and regularized regression was applied, respectively (43). Results from this study indicated that the radiomic model produced by Mukherjee et al. showed strong-to-moderate power in predictive prognosis for patients diagnosed with HNSCC, which was further validated in an external institutional testing cohort. In other words, this study concluded that radiomic CT models have significant value in predicting features typically indicating pathological assessment of HNSCC (43). Many of these pathologic features are specific to the individual regions of the head and neck and will therefore be reviewed by region (**Figure 2**).

**Table 1:** Summary of radiomic applications in head and neck.

Classification	Prediction Target	Radiomic and Clinical Features	Source
Nasopharynx	Progression free survival	Multiparametric MRI features	(37)
	Progression free survival	EBV DNA, Gross tumor volume (GTVnx), lymph node (GTVnd), Dose Volume Histogram	(59)
Oropharynx	HPV status	CT imaging: gross tumor volume (GTV)	(63)
	HPV status	CE-CT imaging: gross tumor volume (GTV): high intensity, small lesions, greater sphericity, heterogeneity	(64)
	Local tumor control status post chemoradiation	CT imaging: shape, intensity, texture, wavelet transformation, heterogeneity, HPV status	(32)
Hypopharynx	Treatment response	PET imaging: surface to volume ratio, spherical disproportion, TGV, local homogeneity, variance	(70)
	Disease progression	CE-CT and NC-CT image features, clinical identification of peripheral Invasion	(71)
Larynx	T category prediction radiomics model	CT imaging: gradient skewness and mean, least axis, sphericity, wavelet kurtosis	(72)
	Overall survival	CT texture features	(73)
	Treatment response	FLT PET tumor heterogeneity	(28)
	Local control	CT imaging: entropy, kurtosis skewness, standard deviation	(74)
Parotid gland	Differentiation of MALToma from benign lymphoepithelial lesion	CT based hybrid radiomic and clinical demographic model	(82)
Metastatic	PDL-1 expression	FDG PET textural features, HPV status, Ki-67 expression	(87)

### Nasopharynx

Typically viewed as an endemic within the southern Chinese population, undifferentiated nasopharyngeal carcinoma (NPC) has the strongest association with EBV infection (57, 58). The World Health Organization (WHO) has characterized NPC into two primary histological types: keratinizing squamous cell carcinoma (Type I) and non-keratinizing squamous cell carcinoma (Type II and III). The undifferentiated histological subtype of NPC, such as Type II and III, has the closest association with EBV infection, which particularly affects regions such as Hong Kong, southern regions of China, and Southeast Asia (58). Additional risks include are genetic predisposition and dietary factors. It is important to note that although EBV infection is discovered in nearly all undifferentiated NPC cases, EBV is not detected in other head and neck cancers, excluding salivary gland tumors (58).

#### Exploring the application of Radiomics to Nasopharyngeal Cancer

In a study performed by Zhang et. al., multiparametric magnetic resonance imaging (MRI)-based radiomics was utilized as a prognostic factor in patients with advanced NPC. For this study, 118 advanced NPC patients were enrolled to determine the training cohort (n = 88) and the validation cohort (n = 30). A total of 970 radiomic features were extracted from two parameters: T2-weighted (T2-w) and contrast-enhanced T1-weighted (CET1-w) MRI images. Application of LASSO regression was utilized to select features for progression-free survival (PFS) nomograms and the association between radiomic features and clinical data was evaluated *via* heatmaps (37). The results indicated that there are significant associations between the radiomic features and PFS. For example, radiomic signatures derived from joint CET1-w and T2-w images displayed improved prognostic performance when compared to signatures derived from the CET1-w and T2-w parameters separately. These findings were confirmed in the validation cohort, suggesting the application of radiomics utilizing multiparametric MRI-based radiomics provided improved prognosis in advanced NPC. Nonetheless, there is a need to research features that can be utilized in radiomic application to profile these types of advanced NPC tumors. Producing these findings will allow for treatment advancement and precise clinical risk stratification (20).

#### Exploring the application of Radiomics to the Epstein-Barr Virus in Head and Neck Cancer

EBV in relation to HNSSC has the strongest association with nasopharyngeal carcinoma (NPC). In a study performed by Yang K. et. al., the study aimed to develop and validate a nomogram that incorporated clinical data, gross tumor volume of the nasopharynx (GTVnx) and lymph nodes (GTVnd) radiomic signatures, and multiparametric based therapeutic dose-volume histogram (DVH) signatures by Least Absolute Shrinkage and Selection Operator (LASSO) to predict progression-free survival (PFS) in patients diagnosed with locoregionally advanced NPC. The study concluded that the developed multidimensional nomogram incorporating radiomic signatures of lymph nodes, planning scores, and tumor-node-metastasis stage showed efficient predictive accuracy in determining PFS. However, incorporating pre-treatment plasma EBV-DNA status improved the predictive accuracy of the nomogram model. This implication was investigated *via* a sub-group analysis of EBV-DNA (59). This data was confirmed by the study’s validation cohort, and as a result, indicated that consideration of pre-treatment EBV-DNA was a useful prognostic biomarker in NPC (59). Therefore, there is potential improvement in NPC screening when considering radiomics and EBV-status.

### Oropharynx

Oropharyngeal cancer (OPC) is one of the most frequent HNC, with squamous cell carcinoma (SCC) accounting for approximately 90% of diagnosed cases (60). The oropharynx is a region in the pharynx located behind the oral cavity, including structures such as the soft palate and tonsils. This cancer has a 5-year-survival rate of approximately 50% (60). The high mortality rate is not always due to the malignancy or intensity of the tumor, but simply due to late detection (60). OPC tumors rarely present symptoms that seem concerning upon initial screening. For example, symptoms typically include a sore throat or difficulty swallowing (60). Therefore, the tumor is usually detected late with little to no time to treat the disease, resulting in low survival rates and death shortly after diagnosis. OPC can also be characterized by its aggressive tumors, with a 70% prevalence of cervical metastases and the ability to disseminate quickly (60). Risk factors for oropharyngeal cancer include a history of smoking cigarettes and the presence of an HPV infection (61).

The association between HPV status and HNSCC involves distinct tumor morphology, younger patient’s age when presented, and positive response to radiotherapy treatment. HPV-positive status is a significant prognostic feature regarding favorable outcomes and overall survival in patients diagnosed with oropharyngeal squamous cell carcinoma (OPSCC) (5). This is because HPV-positivity is considered a strong, independent prognostic feature when diagnosing OPSCC. HPV status of the tumor is determined by analyzing p16 positivity using immunohistochemistry. The cyclin-dependent kinase inhibitor p16 is a tumor suppressor gene that is often overexpressed in HPV mediated cancers and leads to an overall better course of disease (62).

In a study performed by Leijenaar et. al., the study examined that HPV-positive OPSCC is biologically and clinically different than HPV-negative cases. The study then approached understanding these significant differences through radiomics to evaluate the HPV status of OPSCC (63). The study included four independent cohorts that encompassed a total of 778 patients diagnosed with OPSCC. Of the 778 cases, the data was randomly assigned for the radiomic model training (n = 628) and validation (n = 150) cohorts. From pre-treatment CT imaging, 902 radiomic features were extracted from gross tumor volume. Currently, there are no MRI-based radiomic reports available regarding radiomic signature prediction of HPV status.

#### Exploring the Application of Radiomics to Oropharyngeal Cancer

Application of radiomics has been practiced within this field of disease and poses as a promising tool to noninvasively characterize tumor phenotypes (32, 64). In a study conducted by Bagher-Ebadian et.al., a radiomic analysis of primary tumors extracted from pre-treatment contrast-enhanced computed tomography (CE-CT) images was performed on patients diagnosed with OPC (64). Within this study, Bagher-Ebadian et al. utilized radiomics to identify distinct features that construct optimal characterization and prediction of HPV affecting OPC. Amongst the 172 radiomic features that were examined, only 12 radiomic features were significantly different between HPV-positive and HPV-negative patients. Results from this study indicate that gross tumor volumes (GTV) for HPV-positive patients display higher intensity, smaller lesion size, greater sphericity, and higher patient intensity-variation/heterogeneity on CE-CT imaging (64). These results suggest that radiomic features of HPV status in OPC patients are associated with spatial arrangement and morphological appearance *via* CE-CT imaging.

Furthermore, in a retrospective study performed by Bogowicz et al. CT radiomics was utilized to predict local tumor control (LC) after chemoradiation therapy of HNSCC, as well as examining the effects of HPV infection on tumor radiomics. A training cohort (n = 93) and a validation cohort (n = 56) were approved to be included in this study. 317 CT-radiomic features were calculated within the primary tumor region, including features based on shape, intensity, texture, and wavelet transformation (32). Results from this study indicated that 3 features were significantly associated with LC, indicating that tumors with a heterogeneous CT density were at risk for decreased LC (32). As a result, this study concluded that quantified CT radiomics examining the heterogeneity of HNSCC tumor density is associated with LC after chemoradiation therapy and HPV status (32). Utilizing this radiomic information from studies such as Bagher-Ebadian et al. and Bogowicz et al. will allow for clinicians to further optimize oral screening for OPC and HNSCC, therefore optimizing patient diagnosis and clinical decision making in treatment planning.

### Hypopharynx

Hypopharyngeal cancer has the worst prognosis of all HNC with a 5-year-survival of only 25% to 41% (65–67). It is uncommon, with 2,500 new cases arising annually within the United States (68). The hypopharynx can be divided into three distinct regions to better distinguish the localized cancer cells: pyriform sinus, postcricoid region, and the posterior wall (68). The pyriform sinus is where most squamous cell carcinomas occur, with 70% of cases arising within this region. The postcricoid region accounts for approximately 20% of cases and the posterior wall accounts for approximately 10% of cases (69). Because typical presentation is usually recognized by the growth of a neck mass or dysphonia, newly diagnosed patients are often presented at Stage III or IV of disease, contributing to this disease history of poor prognosis (68). Hypopharyngeal cancer typically affects individuals ranging between the ages of 50 to 60 years, occurring more often in men than women. Superior localization of the cancer cells is mostly associated with heavy drinking and smoking. Nutritional deficiencies account for the postcricoid, the inferior part of the hypopharynx, being affected (68). Hypopharyngeal tumors are classified as highly aggressive due to their ability to metastasize early and infiltrate an abundant submucosal lymphatic network, sometimes even skipping metastasis and reappearing in various locations distinct from the primary site. Therefore, it is very common for multiple primary tumors to resurface (68). Treatment of hypopharyngeal cancer is often controversial due to the desire for organ preservation (65, 67). Early detection of this carcinoma may only require radiotherapy, but treatment for later stages of the disease is more complicated. Due to the complications of late-stage disease, the standard treatment is surgical resection and is sometimes paired with postoperative chemoradiation therapy with or without immunotherapy (69).

#### Exploring the Application of Radiomics to Hypopharyngeal Cancer

Since early detection of this disease may only require treatment *via* radiotherapy, identifying significant markers that indicate the carcinogenesis of hypopharyngeal cancers into a non-invasive radiomic pipeline could potentially improve prognosis. Utilizing radiomics may allow clinicians to assess the progression of the disease earlier, and, therefore, to construct a patient-specific treatment plan that optimizes treatment response and reduces unnecessary high-risk intervention. Fortunately, studies have shown that early detection of the tumor can be found using radiomics. Liao et al. conducted a study including a total of 80 OPC and hypopharyngeal cancer PET images were analyzed using radiomics to distinctively select imaging features indicative of the diseases. These imaging features were then correlated with prognostic diagnosis, cancer stage detection, and prediction of effective treatment. All cases included in the study had been treated with chemoradiation therapy (70). This study found that 16 image features were significantly different between early and late stages within the several metabolic tumor volumes (MVT). The image features include surface area, surface to volume ratio, compactness, spherical disproportion, TGV, energy, contrast, local homogeneity, dissimilarity, variance, inverse variance, inverse difference moment, inverse difference, RLNU, and RPC. These features successfully differentiated early from late stages of OPC and hypopharyngeal cancer. As a result, these findings assisted in evaluating prognosis and specific treatment response for the patient (70). 5 and 2 features had an area under curve (AUC) in receiver operating characteristic (ROC) greater than 0.7, indicating a promising predictor. The studied imaging features resulted to prove to be essential indicators in tumor differentiation, staging, overall survival (OS), relapse, and treatment efficacy (70).

Additionally, a study conducted by Mo et al. established a radiomics-based model to classify early versus late detection and metastatic disease in patients with hypopharyngeal cancer. 113 patients diagnosed with this carcinoma were treated with chemoradiotherapy and divided into two cohorts, a training cohort (n = 80) and a validation cohort (n = 33) (71). The radiomics model utilized the concordance index (C-index) to predict prognostic factors, resulting in C-indices of 0.804 with a 95% confidence interval (CI) of 0.688-0.920 and 0.756 with a 95% CI between 0.605-0.907. Furthermore, the log-rank test and a nomogram were used in risk prediction of the model to assess disease progression. The significant results were p=0.00016 and p=0.00063, demonstrating an effective classification of patients into high and low-risk categories (71). Overall, the radiomics model in this study suggests being effective in predicting the risk of progression for hypopharyngeal cancer along with chemoradiotherapy (71).

### Larynx

Laryngeal squamous cell carcinoma (LSCC) consists of 30-50% of all neoplasms in the head and neck (15). Treatment surrounding this disease is difficult due to considerable amounts of variability concerning the region’s anatomy, its surrounding structures, variable appearance of primary and recurrent tumors, significant anatomic changes resulting from tumor response, and high intratumoral heterogeneity (15). Standard-of-care treatment towards LSCC prioritizes organ-preserving strategies, although treatment options may be limited for more aggressive diseases. Although these strategies focus primarily on limiting the functional complications that are associated with complete surgical removal of the larynx, the most appropriate therapy for patients with advanced LSCC is a total laryngectomy (72). Conducting a surgical plan for treatment relies heavily on tumor T categories defined by the National Comprehensive Cancer Network (NCCN) Guidelines (72).

However, relapse occurrence resulting from these organ-preserving treatment approaches remains high, with recurrence at 5-years approximately 30-40%, despite overall improvement in radiotherapy and systemic techniques (15). Exploring the radiomic study of one of the most anatomically complex structures within the head and neck region can provide additional comprehensive information and characterization of intra-tumor heterogeneity.

#### Exploring the Application of Radiomics to Laryngeal Squamous Cell Carcinoma

Surgical options for patients diagnosed with LSCC heavily depend on preoperative T category classification, specifically between T3 and T4 categories. This is because the distinction between T3 and T4 categories for LSCC relies on the destruction degree of the extralaryngeal spread and/or outer cortex of thyroid cartilage (72). However, determining the T category pre-operatively has its clinical challenges due to variable clinical deductions between imaging modalities. Commonly used imaging techniques include CT and MRI, both techniques harboring individual benefits and limitations (72). Therefore, a T category prediction radiomics (TCPR) model that combines radiomic signature and T category distinction could be beneficial in establishing optimal surgical outcomes. A study conducted by Wang et al. was done to further validate the precise prediction of T categories using a radiomic nomogram and the TCPR model to assess appropriate treatment management for each individual case. This study included a total of 211 patients with LSCC who had total laryngectomies separated into two cohorts. The training cohort (n=150) and the validation cohort (n=61) yielded results that demonstrate great capabilities of the TCPR model in predicting the preoperative T categories per patient. The T category resulting from the study has an AUC of 0.775 (95% CI: 0.667–0.883). The radiomic signature resulted in a higher AUC, with AUC 0.862 (95% CI: 0.772–0.952). Finally, the nomogram incorporating the radiomic signature as well as the T category, the TCPR model, resulted in an AUC of 0.892 (95% CI: 0.811–0.974). These results show that the predictive performance of the T category improves with the application of the TCPR model (72).

Moreover, in a study conducted by Chen et al., radiomic analysis of laryngectomy CT imaging of 136 patients with LSCC was performed to assess the prognostic value of radiomics derived from CT. All patients were divided into the training cohort (n = 96) and the validation cohort (n = 40). A method was designed to establish a radiomics signature from the CT texture features and a radiomics nomogram to predict overall survival (OS) (73). The validation of the nomogram was done by a calibration curve, C-index, and decision curve. The results revealed the radiomics signature to have C-indices of 0.782 (95%CI: 0.656–0.909) and 0.752 (95%CI, 0.614–0.891). The radiomics nomogram had outdone the cancer staging capability with a C-index of 0.817 *vs*. 0.682; *P* = 0.009 in the training cohort and a C-index of 0.913 *vs*. 0.699; *P* = 0.019 in the validation cohort (73). The radiomics nomogram has had a significant difference in its discrimination capability when compared to other cancer staging techniques. The calibration and decision curves have been shown to have an accurate prediction for OS as well. This study has successfully utilized radiomics in a way that predicts OS for LSCC, is critical in constructing a personalized treatment plan for each individual patient (73).

In another study conducted by Ulrich et al., radiomic feature analysis from various 18F-fluorothymidine positron emission tomography (FLT-PET) was done to evaluate the prediction of treatment response in patients with HNC. Thirty patients in the late stages of OPC and LSCC who underwent chemoradiation therapy and FLT-PET imaging before surgery were included in the study. 377 radiomic features of FLT uptake were extracted, 9 of which were indicated as significant (28). Within the 30 HNC cases, the study concluded that cases presenting smaller, homogeneous lesions at baseline resulted in a better prognosis. Furthermore, features extracted from the entire lesions had a higher C-index than primary tumor features for the majority of the 9 significant features. Overall, this study has shown that for future studies integrating FLT-PET in predicting prognostic outcome, radiomic features incorporating lesion shape, size, and texture features should be considered to ensure an improved understanding of the disease (28).

Additionally, the increasing application of radiomics to LSCC has demonstrated efficacy in predicting inferior local control and laryngectomy free survival (LFS). A study done by Agarwal et al. explores if pre-treatment CT imaging features of the LSCC can predict long-term local control and LFS. This study analyzed 60 imaging texture features of patients undergoing chemoradiation (CTRT), which were further evaluated with a texture analysis software (74). The data consisted of entropy, kurtosis, skewness, standard deviation, mean intensity, and so on. After a median follow-up of about 24 months, it was found that 39 patients were locally controlled and 10 had been treated with laryngectomy (74). Medium filtered-texture feature that had poor LFS were entropy ≥4.54, (*p* = 0.006), kurtosis ≥4.18; *p* = 0.019, skewness ≤−0.59, *p* = 0.001, and standard deviation ≥43.18; *p* = 0.009). The inferior local control was associated with medium filtered texture features with entropy ≥4.54; p 0.01 and skewness ≤ – 0.12; *p* = 0.02. The analysis of the study has shown medium texture entropy to be a predictor for local control and LFS (*p* = 0.001 & *p* < 0.001). This advancement is undoubtedly efficient in developing prognostic factors for LSCC and predicting treatment response (74).

### Salivary Glands

Salivary gland cancer (SGC) is rare, compromising less than 1% of all cancers in the United States. This type of cancer is prevalent in the older population, mostly affecting individuals between the ages of 50 and 60 (75). The 5-year survival rate of SGC is approximately 7% (76). Residing within the facial region, three major glands are used to classify different types of areas of SGC – the parotid, sublingual, and submandibular glands. Generally, about 80%, 11%, and less than 1% of SGC cases are found within the parotid gland, submandibular gland, and sublingual gland, respectively. Regarding the frequency of malignancy, 20%, 45%, and up to 81% of parotid tumors, submandibular gland tumors, 81% of sublingual gland tumors are malignant, respectively (77). Although there are effective treatments for SGC, successful treatment for sublingual gland cancer is unknown due to lack of clinical trials and the rarity of diagnosis (78). Standard of care treatment typically involves regional surgical resection of the parotid gland, otherwise known as a superficial parotidectomy (77). Although more difficult to treat, cases of malignancy typically require a total parotidectomy. However, this procedure is considered high risk as it involves contact with critical facial nerves that may result in facial paralysis, in more severe cases (77).

### Parotid Gland

Parotid tumors are the most common type of SGC, with the parotid gland accounting for approximately 25% of human saliva. It is the largest salivary gland and resides within the parotid space amongst the external carotid artery, retromandibular vein, and the intraparotid lymph nodes. In some cases, an accessory parotid gland is present on the surface of the masseter muscle (77). The majority of parotid tumors are discovered as benign, though some lesions can be malignant (79). The different cancer subtypes of SGC that can occur in the parotid gland include pleomorphic adenoma, Warthin’s Tumor (War-T), parotid carcinoma (PCa), and Kimura’s Disease (KD) (80). The most common of the subtypes is pleomorphic adenoma. Pleomorphic adenoma composes of epithelial cells along with myoepithelial cells, which are commonly referred to as benign mixed tumors (BMT) (81). Factors that may cause carcinogenesis of pleomorphic adenoma include irradiation, dehydration, and tobacco use (81).

#### Exploring the Application of Radiomics to Parotid Tumors

Regarding parotid tumors, one study implored radiomics to improve diagnostic efficacy and, therefore, treatment options. To improve differentiation of a benign lymphoepithelial lesion (BLEL) and a malignant mucosa-associated lymphoid tissue lymphoma (MALToma) in the parotid gland, Y.-M. Zheng et al. developed a CT-based radiomics nomogram that integrated the radiomics signature alongside clinical data such as demographics (82). This integrated model was trained (*n=70*) and validated (*n=31*) on a total of 101 patients with BLEL or MALToma (82). In developing this model, 851 radiomics features extracted from CT images were narrowed down to 7 features by removing features with poor inter- and intra-observer agreement between radiologists, including features that showed significant differences between BLEL and MALToma (p < 0.000 to 0.050) and applying LASSO regression (82). After performing a multiple logistic regression analysis, statistically significant clinical factors of age (*p* = 0.0036*)* and maximum diameter (*p* = 0.019) were integrated with the radiomics signature resulting from the 7 radiomic features to produce a CT-based radiomics nomogram that showed a statistically significant difference between BLEL and MALToma (82).

### Submandibular Gland

The submandibular gland is the second largest salivary gland. This gland accounts for 70% of human saliva and is located underneath the jawbone (79). Despite the rarity of tumors in the submandibular gland compared to the parotid gland, the probability of malignancy in the submandibular gland is approximately 43% and results in a poorer prognosis (83). Due to rarity and high rates of malignancy, there is a lack of knowledge pertaining to treating submandibular gland tumors (83). There are no definitive treatments for submandibular tumors, but there are numerous ways that have been proven to be successful – all involving high-risk surgery. A common procedure that is performed is submandibular sialoadenectomy, which is to surgically remove the submandibular gland in its entirety (84). The efficacy of radiotherapy in targeting these mass neoplasms is not well known with this type of cancer and is still being evaluated. Chemotherapy in general is not shown to be successful in treating submandibular gland tumors but is sometimes used for treatment if the tumor progressively spreads within the gland (83).

#### Exploring the Application of Radiomics to Submandibular Tumors

In general, there remains uncertainty due to a lack of knowledge for treatment of these diseases, demonstrating the necessity of exploratory measures. Radiomic application to diseases such as submandibular gland cancer illuminates characteristics that can be extracted into operational data. This data can then be utilized to improve detection and lead the course of treatment when managing this disease.

### Sublingual Gland

Sublingual salivary gland tumors are the rarest tumors found in SGC. The sublingual gland is the smallest of the three major glands, residing just below the floor of the mouth and is positioned under the tongue, producing 5% of human saliva (79). Sublingual salivary gland tumors typically affect individuals between 50 to 60 years old and are not specific to gender (85). Sublingual gland tumors are typically malignant, boasting an 81% probability of malignancy associated with this disease type. Adenoid cystic carcinoma and mucoepidermoid carcinoma are the most common neoplasms found in the sublingual gland. Prognosis for adenocarcinoma of the sublingual gland relies on the histology of the specific tumor. This tumor is commonly misinterpreted as minor salivary gland tumors or other malignant lesions within the mouth due to its compact mass (85). Patients normally present no symptoms, making the tumor difficult to identify and accurately diagnose. When evaluating the tumor, it is important to distinguish if it lies in the sublingual gland or any of the minor salivary glands. This cannot be done solely based on location on anatomy, but from a collection of imaging, surgical, and clinical data to ensure accurate diagnosis (85).

#### Exploring the Application of Radiomics to Sublingual Gland Tumors

Due to the rare nature of sublingual glands, specific suggestions for treatment have not been developed, the lack of radiomic studies. However, proper diagnosing of malignant sublingual glands from other types of malignancies has been a challenge (85). Although advances in diagnostic imaging technology have helped with more effective identification, malignant sublingual glands vary in degrees of malignancy and lead to difficulties in not only diagnosis but also management and treatment (85). Radiomics has the potential to improve the initial evaluation of malignant gland tumors since there is a recurrence rate of 50% for these tumors (85).

### Radiomic Application to Advanced Head and Neck Cancer

The management of metastatic and locally advanced head and neck cancer has changed dramatically in the last several years. Keynote 048 was a landmark trial that resulted in FDA approval for the use of immunotherapy either alone or in combination with platinum-based chemotherapy as a first line treatment (78). Specifically, this trial evaluated the efficacy of pembrolizumab, an immune checkpoint inhibitor that allows cytotoxic T cells to recognize programmed death ligand 1 (PDL-1) overexpressed by tumor cells, resulting in their destruction (78). In general, PDL-1 expression by the tumor is evaluated by immunohistochemistry and serves as both a prognostic indicator and as a variable in the decision-making process when selecting an appropriate immunotherapy regiment. The application of radiomics has further potential of evaluating the predictive power of PDL-1 expression, and overall patient outcomes.

While the radiomics of PDL-1 expression has been studied in other tumors such as non-small cell lung cancer, data on radiomic PDL-1 expression in head and neck cancer is lacking (86). One pilot study by Chen et al. was able to predict PDL-1 expression through FDG PET (87). This was accomplished by dichotomizing other biomarkers such as HPV status (p16 positivity) and Ki-67 expression. Textural features were also used to predict PDL-1 expression. For example, gray-level nonuniformity for run (GLNUr), run percentage (RP), and short-zone low gray-level emphasis (SZLGE) were inversely proportional with PDL-1 expression. While it is promising to see evidence of the predictive power of PDL-1 expression afforded by radiomics, this study is limited by its small cohort size. Further studies are needed to reproduce results and optimize the parameters relevant to head and neck cancer.

## Thyroid Cancers

Defined as a malignancy of the thyroid gland by the International Classification of Diseases, Tenth Revision (ICD-10), thyroid cancer accounts for 3.8% of all cancers in the United States and has a five-year survival of 98.3 (88). Thyroid cancers include 3 main types: differentiated thyroid cancer (DTC), anaplastic thyroid cancer (ATC), and medullary thyroid cancers (MTC) (89). Included in DTC, which accounts for over 90% of all thyroid cancers, are papillary thyroid cancer (PTC), follicular thyroid cancer, Hurthle cell, and poorly differentiated thyroid cancer (PDTC) (89). ATC accounts for less than 2% of call thyroid cancers, and MTC accounts for about 1%-2% of all thyroid cancers in the United States. Both DTC and MTC generally have good prognoses, with a 10-year survival rate of 80–95% for PTC, 70–95% for follicular thyroid cancer, and 96% for MTC (90, 91). However, ATC does not share such numbers, as it has a 5-year survival rate of 0-10%. Due to its rare and highly aggressive nature, ATC requires a multidisciplinary team approach with different treatment options of surgery, chemotherapy, or tracheotomy (89). Surgical resection is the standard of care treatment option for DTC and MTC (89).

### Radiomic Application to Thyroid Cancers

There is a need for establishing a non-invasive assessment technique that allows for the mapping of thyroid tumors in their entirety. It is important to expand the knowledge of radiomics and explore its implication to various disease types to improve clinical diagnosis and patient’s quality of life. According to a study performed by Liang et. al., application of radiomics showed good performance and potentially outperformed ACR TI-RADS (American College of Radiology, Thyroid Imaging, Reporting, and Data System) scoring when predicting the malignancy of thyroid nodules (92). The objective of this study was to produce a radiomic score utilizing US imaging to predict the probability of malignancy in thyroid nodules when compared to the ACR TI-RADS criteria. To do so, pathologically proven thyroid nodules were enrolled to produce a training cohort (one hospital, n=137) and a validation cohort (separate hospital, n=95). The radiomic score was developed utilizing the training cohort. US images were reviewed by two junior and one senior radiologist and scored the nodules based on the 2017 ACR TI-RADS scoring criteria (92). Results from this study indicated that the radiomic score had good discrimination, with an AUC of 0.921 in the training cohort and 0.931 in the validation cohort. This result suggests that the radiomic score was significantly more accurate than the ACR scores when scoring suspicious thyroid nodules (**Table 2**). As a result, a decision curve analysis showed that the radiomics score model potentially added more benefits than using the ACR TI-RADS scoring criteria (92).

**Table 2:** Summary of radiomic applications in thyroid cancer.

Category	Prediction Target	Radiomic Features and Clinical Information	Source
Thyroid nodules	Malignancy	US Thyroid radiomic score	(92)
Papillary Thyroid Cancer	Progression free survival	US Thyroid: tumor size, cervical lymphadenopathy, extrathyroidal extension, gray level scores	(25)
Follicular Thyroid Cancer	Metastatic disease	US Thyroid: tumor shape, gray level scores	(97)
Medullary Thyroid Cancer	Treatment response to PRRT	SSTR- PET: textural features (gray level non uniformity)	(101)
Anaplastic Thyroid Cancer	Treatment response/dose adjustment of Trametinib	Radiolabeled Trametinib	(105)

### Papillary Thyroid Cancer

Papillary thyroid cancer (PTC) is the most diagnosed thyroid cancer, accounting for approximately 80% of well-differentiated thyroid cancers. Although PTC typically has favorable outcomes and a mortality rate of 1.2% at 20 years, patients with recurrent disease have poorer outcomes. Approximately 10% to 15% of PTC cases recur, resulting in 35% of these patients ultimately dying from this cancer. This is because recurrent PTC patients present aggressive features such as extrathyroidal extension (ETE), aggressive pathological cell subtypes, the extent lymph node involvement, resistance to therapeutic treatments, and distant metastasis (93). To assess these aggressive features, clinicians use a variety of techniques such as ultrasound and ultrasound-guided fine-needle aspiration to develop a diagnosis. An additional imaging modality that is often utilized is MRI. This allows for superior contrast of the soft tissues when examining the thyroid region, affording assessment of aggressive features such as ETE and neck nodal metastasis (93, 94). Although these imaging modalities are standard-of-care practices, both harbor limitations in accuracy and therefore inhibit optimal clinical assessment of the disease.

#### Exploring the Application of Radiomics to Papillary Thyroid Cancer

In a retrospective study conducted by Park et. al., the association between a radiomic signature of conventional ultrasound (US) images and disease-free survival in PTC was investigated. The history of this disease type shows that PTC is considered a “good cancer” with regards to its treatability and relatively favorable survival rate (25). However, there is a small amount of PTC cases that show clinically aggressive behavior that results in 9% to 13% of patients experiencing recurrence and 1% to 5% of patients ultimately dying from thyroid cancer. Considering this information, patients diagnosed with aggressive PTC would greatly benefit from radiomic application with a preoperative risk stratification tool that assists in assessing treatment plans and follow-up procedures (25).

### Follicular Thyroid Cancer

Follicular thyroid cancer (FTC) is known as the second most common differentiated thyroid cancer, accounting for 10% to 15% of all cases. When considering age and gender, this disease subtype typically affects women 50 to 60 years old. FTC presents more aggressively in comparison to PTC, as this disease typically invades blood vessels and is capable of metastasizing *via* hematogenous dissemination. Knowing this information, FTC is associated with a poorer prognosis in comparison to PTC, as FTC patients often present with more advanced staging of disease due to vascular invasion (95). Long-term survival rates in patients diagnosed with metastatic FTC range between 31% to 43%, taking into consideration the patient’s age at the time of diagnosis, tumor size, capsular invasion, gender, and evidence of metastases (96). FTC is typically classified into two categories: minimally invasive or widely invasive.

#### Exploring the Application of Radiomics to Follicular Thyroid Cancer

In a study conducted by Kwon et. al, radiomics was utilized to evaluate distant metastasis of FTC on gray-scale US images. This retrospective study included 35 cases of FTC with distant metastases and 134 cases of FTC without distant metastasis (97). A total of 60 radiomic features were extracted, deriving from the first order, shape, gray-level co-occurrence matrix, and gray-level size zone matrix features utilizing US imaging techniques. Results from this study indicated that the support vector machine (SVM) classifier had an AUC of 0.90 on average on the test folds (97). Radiomic signature (p<0.01) and widely invasive histologies (p = 0.003) proved to be significant when associated with distant metastasis on multivariate analysis (97). From multivariate analysis, the SVM classifier indicated an AUC of 0.93. As a result, this study indicated that utilizing radiomic signatures from thyroid US can be an independent biomarker in order to non-invasively predict the probability of distant metastasis of FTC (97). However, this study does harbor limitations. It primarily lacks external validation, as the study was performed at a single institution. Additionally, FTC with distant metastasis is considered rare, naturally limiting the study. As a result, it is necessary to further validate radiomic application amongst different variables in FTC in order to successfully translate radiomics to FTC diagnosis.

### Medullary Thyroid Cancer

Medullary thyroid carcinoma (MTC) derives from the calcitonin-secreting parafollicular C cells of the thyroid, accounting for up to 1% to 3% of all malignant thyroid cancer cases (98–100). Two forms of MTC currently exist: sporadic and hereditary. The hereditary form of MTC is expressed in an autosomal dominant fashion caused by a mutation of the receptor tyrosine kinase (RET) proto-oncogene (99). This mutation causes hereditary MTC to be associated with diseases such as multiple endocrine neoplasia 2 (MEN 2) syndrome (98, 99). This subtype of hereditary MTC can be further characterized as MEN2A and MEN2B. MEN2A presents in approximately 80% of inherited MTC cases, showing symptoms such as multifocal and bilateral MTC, pheochromocytoma, and primary hyperthyroidism (99). MEN2B presents in approximately 5% of inherited MTC cases and is associated with pheochromocytoma, multiple mucosal neuroma, and Marfan syndrome (98, 100). An additional subtype of MTC is known as familial MTC (FMTC) and is diagnosed in patients that have a family history of MTC which have at least four family members diagnosed with MTC with no history of pheochromocytoma or primary hyperthyroidism (98, 99). MTC often presents as a poor prognosis with early lymph node metastasis, aggressive invasiveness of key surrounding organs, and failure to respond to radiation therapy and/or chemotherapy. As a result, early detection and preventative surgery is often the standard-of-care treatment plan regarding MTC (98).

#### Exploring the Application of Radiomics to Medullary Thyroid Cancer

Regarding medullary thyroid cancers, there is great potential for radiomics to be utilized here. One study shows promise in improving prognosis by exploring radiomic features involved with PET images of advanced medullary thyroid cancer (101). Lapa et al. assessed tumor heterogeneity by investigating the association between textural parameters on somatostatin receptor PET (SSTR-PET) and treatment response to peptide receptor radionuclide therapy (PRRT) on 4 medullary thyroid cancer patients and 8 radioiodine-refractory differentiated thyroid cancer patients (101). They found that several textural parameters showed a significant capability to assess PFS, with “grey level non uniformity” ranking with the highest AUC (0.93) in ROC curve analysis and “contrast” with the ranking second highest AUC (0.89) (101). Further assessment of other radiomics features might assist in considering PRRT as a treatment option for patients.

### Anaplastic Thyroid Cancer

Anaplastic thyroid cancer (ATC) is the rarest and most aggressive of the thyroid cancer subtypes, accounting for 1% to 2% of all thyroid malignancies. Although incidence is rare, diagnosis of this subtype results in over 50% of deaths from thyroid cancer with a median survival of only six months (102). Amongst all malignancies, ATC is a highly aggressive disease with one of the worst prognoses due to its resistance to standard therapies and management difficulties (102). ATC has been known to arise in two forms: *de novo* or by dedifferentiation from a well-differentiated thyroid cancer such as PTC (103). Standard-of-care treatment is typically surgical resection of the cancerous lesion, followed by adjuvant radiotherapy and/or chemotherapy (104).

#### Exploring the Application of Radiomics Anaplastic Thyroid Cancer

Due to anaplastic thyroid cancer’s aggressive nature and poor prognosis, there is a major lack of radiomic studies on it. However, utilizing radiomics can help predict resistance to an FDA approved therapy for ATC – trametinib (105). Trametinib is a highly potent, efficacious, yet toxic, treatment option for ATC, so modifying the dose is desirable (105). In a study conducted by Pratt et. al., a radiolabeled version of trametinib, 124I-trametinib was developed to potentially assess therapeutic index and personalize individual doses for patients (105).

### Parathyroid Cancer

Parathyroid carcinoma (PC) is a less common cancer, diagnosed in <1% of cases within primary hyperparathyroidism (PHPT). Although this disease is generally seen as sporadic, it may appear in familial PHPT, specifically within hyperparathyroidism-jaw tumor syndrome (HPT-JT). Extremely rare cases of PC may arise from multiple endocrine neoplasia type I (MEN1) (106). It is difficult to diagnose PC preoperatively because this disease type has a lack of specific biochemical and clinical features (106). As a result, this disease is typically diagnosed postoperatively when the disease is being examined histologically and/or when the disease recurs (106).

#### Exploring the Application of Radiomics to Parathyroid Cancer

Although there are no studies on the application of radiomics to parathyroid cancer, there is a need for clinicians to be able to differentiate between parathyroid adenoma (benign) and parathyroid carcinoma because of the lack of specific biochemical and clinical features (106). CT and MRI can both help accurately localize the primary tumor, so the use of radiomics shows great promise in the parathyroid glands in PC (106).

## Discussion/Conclusion

Machine learning and deep learning models have been widely used for medical imaging research (6, 107). Although having impressive predictive performance, these models are often difficult to interpret. Additionally, there may be hidden bias in the model leading to potential ethical issues (108, 109). Interpretability of predictive models has become one of the key factors driving their adoption in clinical decision support environment. To ease the tension between the model prediction accuracy and interpretability, various approaches have been proposed to generate intuitive interpretations of predictive models (110–113).

Radiomic studies are often exploratory in nature. They are normally single institutional with limited cohort size. The associated imaging data are typically acquired from just one or a few scanners from a single site. To deploy radiomic predictive models at scale and possibly across institutions, we need to address issues of potential data variability caused by scanners from different vendors (114), and whether the models are still predictive when they are applied to a different cohort from an external site with similar disease types In summary, being able to standardize image data acquisition and quality control using phantoms, various calibration techniques, having large cohorts from multiple locations for model training, and validation will provide more confidence for deployment in clinical settings.

The application of radiomics to HNC and thyroid cancers is an advancement that allows for a deeper interpretation of a patient’s digital medical imaging data beyond visual assessment. Utilizing this practice, especially in cancer domains that lack radiomic studies such as anaplastic thyroid cancer and parathyroid cancers, will allow for more personalized and patient-specific cancer treatment. By gathering additional statistical data and conducting subsequent analysis, clinical decision making is improved and therefore affects patient outcomes Court, Fave (115).

## Author Contributions

MG, K-JB, DG, CWH, AS, VL, AF, AC, MI, and AAC contributed in literature search and manuscript preparation. MG and AAC performed final edits and revisions. All authors contributed to the article and approved the submitted version.

## Funding

The study was support by NIH grant # 2K12CA001727-26.

## Conflict of Interest

The authors declare that the research was conducted in the absence of any commercial or financial relationships that could be construed as a potential conflict of interest.
